# Overlapping Antisense Transcription in the Human Genome

**DOI:** 10.1002/cfg.173

**Published:** 2002-06

**Authors:** M. E. Fahey, T. F. Moore, D. G. Higgins

**Affiliations:** Department of Biochemistry University College Cork Lee Maltings Prospect Row Cork Ireland

## Abstract

Accumulating evidence indicates an important role for non-coding RNA molecules in eukaryotic cell regulation. A small number of coding and non-coding overlapping antisense transcripts (OATs) in eukaryotes have been reported, some of which regulate expression of the corresponding sense transcript. The prevalence of this phenomenon is unknown, but there may be an enrichment of such transcripts at imprinted gene loci. Taking a bioinformatics approach, we systematically searched a human mRNA database (RefSeq) for complementary regions that might facilitate pairing with other transcripts. We report 56 pairs of overlapping transcripts, in which each member of the pair is transcribed from the same locus. This allows us to make an estimate of 1000 for the minimum number of such transcript pairs in the entire human genome. This is a surprisingly large number of overlapping gene pairs and, clearly, some of the overlaps may not be functionally significant. Nonetheless, this may indicate an important general role for overlapping antisense control in gene regulation. EST databases were also investigated in order to address the prevalence of cases of imprinted genes with associated non-coding overlapping, antisense transcripts. However, EST databases were found to be completely inappropriate for this purpose.


**Comparative and Functional Genomics**
*Comp Funct Genom* 2002; **3**: 244–253.
Published online 2 May 2002 in Wiley InterScience (www.interscience.wiley.com). **DOI:** 10.1002/cfg.173

**Short Communication**

### Overlapping antisense transcription in the human genome

M. E. Fahey, T. F. Moore and D. G. Higgins*
*Department of Biochemistry, University College Cork, Lee Maltings, Prospect Row, Cork, Ireland*

*Correspondence to: Department of Biochemistry, University College Cork, Lee Maltings, Prospect Row, Cork, Ireland.
E-mail: d.higgins@ucc.ie

Received: 31 January 2002
Accepted: 11 April 2002

### Abstract

**Accumulating evidence indicates an important role for non-coding RNA molecules in eukaryotic cell regulation. A small number of coding and non-coding overlapping antisense transcripts (OATs) in eukaryotes have been reported, some of which regulate expression of the corresponding sense transcript. The prevalence of this phenomenon is unknown, but there may be an enrichment of such transcripts at imprinted gene loci. Taking a bioinformatics approach, we systematically searched a human mRNA database (RefSeq) for complementary regions that might facilitate pairing with other transcripts. We report 56 pairs of overlapping transcripts, in which each member of the pair is transcribed from the same locus. This allows us to make an estimate of 1000 for the minimum number of such transcript pairs in the entire human genome. This is a surprisingly large number of overlapping gene pairs and, clearly, some of the overlaps may not be functionally significant. Nonetheless, this may indicate an important general role for overlapping antisense control in gene regulation. EST databases were also investigated in order to address the prevalence of cases of imprinted genes with associated non-coding overlapping, antisense transcripts. However, EST databases were found to be completely inappropriate for this purpose.** **Copyright © 2002 John Wiley & Sons, Ltd.**

**Keywords:** **OAT; antisense transcript; imprinting; human genome; EST; cDNA**

### Introduction

There is accumulating evidence that a large number of structurally and functionally diverse non-coding RNA (ncRNA) molecules are produced in the eukaryotic cell (Eddy, 2001; Mattick, 2001). The hitherto unsuspected complexity of RNA-based gene regulatory mechanisms presents a considerable technical challenge to both bioinformaticists and molecular biologists. Most of the transcripts do not currently have well-defined structural features and may not be represented, or may be dismissed as cloning artifacts, in gene expression libraries. For example, functional ncRNA occurs in sizes ranging from 21 nucleotides for 'small temporal RNA' (stRNA) and double-stranded silencing RNA (siRNA) (Pasquinelli *et al.*, 2000; Harborth *et al.*, 2001), to greater than 40 kb for overlapping antisense or intergenic transcripts associated with chromatin remodeling at imprinted gene loci such as *IGF2R* and *XIST* (Wutz *et al.*, 1997; Lee *et al.*, 1999), and at developmentally regulated, non-imprinted loci such as $\alpha$ globin (Gribnau *et al.*, 2000). Moreover, it is unclear whether a low level of transcription of certain ncRNAs is functionally significant, or whether it merely represents 'illegitimate' or 'leaky' transcription from cryptic promoters.

These difficulties notwithstanding, evidence that ncRNAs make a significant contribution to eukaryotic cell function comes from a variety of sources. Established work indicates that, in addition to functional intronic RNA, ribosomal RNA (rRNA), transfer RNA (tRNA), and the 5′ and 3′ untranslated regions (UTR) of messenger RNA (mRNA), short ncRNAs are also integral components of major nuclear catalytic complexes, for example, the small nuclear RNAs (snRNAs) of the spliceosome (Valadkhan and Manley, 2001), and telomerase RNA (Lukowiak *et al.*, 2001). In addition, a wide variety of transcriptional and translational regulatory mechanisms have been either described or

Copyright © 2002 John Wiley & Sons, Ltd.

proposed that involve the base-pairing of complementary RNA molecules produced either in *cis* or in *trans*. These include the small nucleolar RNAs (snoRNAs), which modify rRNA and snRNAs (Kiss, 2001), the 'microRNAs' (miRNAs) including stRNA and siRNA (Eddy, 2001), and overlapping antisense transcripts (OATs) produced in *cis* at protein-coding gene loci in mammals (Kumar and Carmichael, 1998; Vanhée-Brossollet and Vaquero, 1998).

Imprinted genes, which are expressed from only one of the parental alleles during mammalian development, comprise a functionally diverse family of developmentally regulated genes with unusual genomic features, such as associated tandem repeats (Neumann *et al*., 1995) and reduced intronic content (McVean *et al*., 1996). In addition, there may be an enrichment of OATs at imprinted loci (Moore, 2001). Such imprinted, antisense transcripts may be functionally significant because many are expressed at high levels and are associated with genomic regions implicated in regulating the imprinting mechanism (Moore *et al*., 1997; Sleutels *et al*., 2000). However, there are also examples of OATs at non-imprinted loci (Vanhée-Brossollet and Vaquero, 1998). Moreover, the apparent enrichment of such transcripts at imprinted loci may reflect an ascertainment bias, because of the intensive study of the genomic organization and allele-specific expression patterns of these genes relative to non-imprinted genes.

In order to address the question of the functional significance of OATs in the human genome, we sought to estimate the frequency of their occurrence and to delineate their genomic structures through bioinformatics, by using BLASTN to search for sequence complementarities between transcribed gene sequences in the public databases. We were able to place a lower boundary on this estimate of approximately one thousand OATs in the human genome.

### Materials and methods

The RefSeq database (Pruitt *et al*., 2001), which contains annotated mRNA sequences for 11 015 different human genes (at January 2001), was used. These are high quality gene predictions that use a combination of the scientific literature, expressed sequence tag (EST) sequences and automatic predictions of the locations of introns and exons. We downloaded the complete processed mRNA sequences for all genes (**ftp://ftp.ncbi.nlm.nih.gov/refseq/**). These sequences include the coding regions as well as the 5′ and 3′ untranslated regions (UTRs) of each gene. The BLASTN program (version 2.0.13, Altschul *et al*., 1990) was then used to compare each sequence in RefSeq to the complementary strand of all the remaining genes. This locates pairs of genes that have, in principle, the ability to form stretches of double-stranded RNA. The threshold E-value was set to $10^{-8}$ to exclude weak matches. This yielded a collection of 1221 high scoring pairs (HSPs). These included matches due to the presence of repeated sequences (e.g. ALU repeats in the UTRs), which were filtered manually using Repeatmasker (A.F.A. Smith and P. Green, RepeatMasker at **http://ftp.genome.washington.edu/RM/RepeatMasker.html** Smith and Green, 27). The remaining pairs of sequences were then checked using the Locuslink records from RefSeq and the UCSC human genome browser (**http://genome.ucsc.edu/**) to locate pairs of overlapping genes that map to the same chromosomal location.

In a second series of experiments, the sequences of known imprinted genes from human and mouse were examined for complementary matches against corresponding databases of EST sequences using the Gene2est server at **http://www.woody.embl-heidelberg.de/gene2est** (Gemund *et al*., 2001). The list of mouse and human imprinted genes was taken from the Genomic Imprinting Website (**http://www.geneimprint.com/**). The Gene2est server produces a BLAST output, which was imported into Artemis for visualization of results (Rutherford *et al*., 2000). In order to check the validity of EST 'hits' to the complementary strand, mouse RefSeq was blasted against mouse EST sequences from Genbank 124 (June 2001).

### Results

The initial 1221 HSPs from the BLASTN searches were taken and reduced to 56 pairs of overlapping genes as described in Materials and Methods (Table 1). As expected under an assumption of random distribution, a large proportion of the transcripts map to the two largest chromosomes, 1 and 2. The majority of overlaps are between the 3′ UTRs of the transcripts (Table 2), with a smaller number located in the 5′ UTRs, or between the 5′ UTR of one transcript and the 3′ UTR of another. The overlaps typically extend over 50 – 200

Copyright © 2002 John Wiley & Sons, Ltd.

Table 1. A list of overlapping transcripts reported in chromosomal order. The length of the overlap between each pair of transcripts is denoted in nucleotides. The type of overlap is denoted as: between the 3′ UTRs (3′), between the 5′ UTRs (5′), or the 5′ UTR of one transcript and 3′ UTR of the other (3′/5′). If a coding region is involved, it is marked cds. ** denotes a reviewed RefSeq sequence which has been manually processed; * indicates a provisional sequence on which some initial quality checking has been done; the remaining cases correspond to automatically generated predicted records which are validated by cDNA or EST data, and/or closely related homologous sequences

| Name | Map Position | Overlap | Size of Overlap in Nucleotides | Comment |
|---|---|---|---|---|
| *MUF1* | 1p33 | 3′ | 72 | -leucine repeat |
| **RAD54L* | | | | -role in DNA recombination and repair |
| *FLJ20580* | 1p32.3 | 3′ | 94 | -function unknown |
| ***CPT2* | | | | -enzyme of long-chain fatty acid oxidation. |
| *ATP1B1*[1] | 1q24.2 | 3′ | 184 | -beta 1 subunit of Na+/K+ −ATPase |
| **NME7* | | | | -kinase, may be involved in the synthesis of CTP, GTP, and UTP |
| ***FMO1* | 1q24.2 | cds/5′ | 92 | -found in fetal liver, NADPH-dependent flavoenzymes |
| *PRO1257* | | | | -function unknown |
| * *TPR* | 1q31.1 | 3′ | 199 | -provisional, translocated promoter region (to activated MET oncogene) |
| ***PRG4* | | | | -reviewed, Megakaryocyte stimulating factor secreted proteoglycan, |
| *LOC51611* | 1p21.1 | cds+3′/5′+cds | 429 | -function unknown |
| *FLJ20139* | | | | -function unknown |
| **OPN3* | 1q43 | cds+3′/3′ | 1095 | -extra-retinal opsin, G-protein linked receptor |
| **KMO* | | | | -catalyzes the hydroxylation of kynurenine to 3-hydroxykynurenine |
| *KIAA0764*[2] | 2p23.2 | 5′ | 112 | -function unknown |
| **FLJ10624* | | | | -solute carrier family 4 (anion exchanger), member 1, adapter protein |
| *CRIPT* | 2p21 | all/3′ | 1063 | -postsynaptic protein |
| *LOC51088*[3] | | | | -lymphocyte activation-associated protein. |
| **MSH6* | 2p16 | cds+3′/3′ | 177 | -G/T mismatch-binding protein |
| *VIT1* | | | | -function unknown |
| **VRK2* | 2p16.3 | cds+3′/cds+3′ | 457 | -serine/threonine kinase |
| *FLJ10335* | | | | -function unknown |
| ***MTHFD2* | 2p12 | cds+3′/3′ | 92/92+76/76 | -intracellular pH regulation and sodium bicarbonate transport |
| **NBC4* | | | | -involved in initiation of mitochondrial protein synthesis |
| **SSB*[4] | 2q31.1 | cds/3′ | 63 | -binds and stabilizes histone mRNA, acts in maturation of tRNAs |
| *HSPC133* | | | | -function unknown |
| *PRO2900* | 2q37.3 | 3′ | 221 | -mediates cholesterol removal |
| **HDLBP* | | | | -function unknown |
| ***RAF1*[5] | 3p25.2 | 3′ | 105 | -kinase – phosphorylates substrates involved in regulating apoptosis, |
| *HSPC070* | | | | -function unknown |
| ***OGG1vi*[6] | 3p25.3 | 3′/3′ | 56/56 | -involved in base excision DNA repair and removal of 8-oxyguanine |
| ***CAMK1* | | | | -involved in Ca(2+)-regulated processes; member of kinase family |
| *HEMK* | 3p21.31 | 5′ | 64 | -function unknown |
| **LOC51161* | | | | -g20 protein |
| ***FGF2* | 4q26 | 3′/cds+3′ | 583/587+56/56 | -mitogenic, angiogenic, and neurotrophic factor |
| * *NUDT6* | | | | -FGF-AS |
| ***POLR2B* | 4q12 | 3′ | 91 | -subunit of RNA polymerase |
| **IGFBP7* | | | | -may bind to and modulate insulin-like growth factor activity |
| **ARTS-1* | 5q15 | 3′ | 132 | -type 1 tumor necrosis factor receptor |
| **CAST* | | | | -an inhibitor protein of calpain |
| **LOC51306* | 5q31.1 | cds+3′/cds+3′ | 263 | -potential nuclear protein -GAP-like protein |
| * *PKD2L2* | | | | -member of the polycystin family; expressed in testis |
| ***DOM-3* | 6p21.32 | 5′+cds/5′ | 124 | -function unknown, ubiquitous expression and conservation in eukaryotes, |
| ***STK19* | | | | -serine/threonine kinase – function unknown; possibly involved in transcriptional regulation. |
| **PPP2R5D* | 6p21.1 | 3′ | 111 | -regulatory subunit B of protein phosphatase 2, delta isoform |

Copyright © 2002 John Wiley & Sons, Ltd. 

Table 1. Continued

| Name | Map Position | Overlap | Size of Overlap in Nucleotides | Comment |
|---|---|---|---|---|
| *MEA | | | | -male-enhanced antigen; may play a role in mammalian spermatogenesis and/or testis development |
| LOC51106 | 6q25.3 | cds/3′ | 300 | -function unknown |
| *TIAM2 | | | 201 | -guanine nucleotide exchange factor |
| *ASK | 7q21.12 | 5′ | 102 | -activator of CDC7 S phase kinase; required for the G1/S transition |
| *LOC55972 | | | | -mitochondrial carrier family protein |
| **AP4M1 | 7q22.1 | 3′ | 101 | -involved in the recognition and sorting of cargo from the trans-golgi network to the endosomal-lysosomal system. |
| *TAF2E[7] | | | | -component of the TFIID complex; interacts with general transcription factors |
| **KDELR2 | 7p22.1 | 5′+cds/cds | 105 | -retrieves proteins from the Golgi for retrograde transport to the ER |
| FLJ20306 | | | | -function unknown |
| *MEST | 7q22.1 | 3′ | 48 | -mesoderm specific protein |
| *COPG2 | | | | -protein related to gamma-COP; may play a role in cellular vesicle traffic |
| FLJ20530 | 8q22.1 | 3′ | 48 | -function unknown |
| *CCNE2 | | | | -cyclin E2 – functions specifically during the G1-phase of the cell cycle |
| **NPR2 | 9p13.3 | 3′ | 309 | -guanylate cyclase activity |
| *SMP-1 | | | | -sperm associated antigen 8 |
| **TESK1 | 9p13.3 | 3′ | 54 | -serine/threonine kinase – testicular germ cell-specific expression and developmental pattern of expression in mouse. |
| *CD72 | | | | -cell surface protein expressed exclusively on B cells; may be involved in control of B cell proliferation |
| HT009 | 10p15.3 | 3′/5′+cds | 173 | -uncharacterized hypothalamus protein |
| **IDI1 | | | | -involved if formation of cholesterol. |
| *RIG | 11p15.2 | 3′ | 295 | -regulated in glioma |
| *DKK3 | | | | -dickkopf (Xenopus laevis) homolog 3; related to proteins that antagonize Wnt signaling |
| FLJ20539 | 11q12.3 | 3′ | 72 | -function unknown |
| *PHT2 | | | | -member of the proton-dependent oligopeptide transport family |
| *RAD9 | 11q13.3 | 3′ | 205 | -may function as a cell cycle checkpoint protein |
| *PPPICA | | | | -regulates mitosis, putative tumor suppressor |
| *IL18BP | 11q13.4 | 3′ | 668 | -inhibitor of the early Th1 cytokine response; |
| *NUMA1 | | | | -structural component of the nucleus; predicted role in nuclear reassembly |
| *PAF65A | 11q13.1 | cds/3′ cds 5′ | 780/781 | -regulates transcription, cell cycle progression, and differentiation |
| FLJ11136[8] | | | 134/134 | -function unknown |
| **APAF1 | 12q23.1 | 3′ | 57 | -functions in the mitochondrial apoptotic pathway that leads to caspase 9 dependent activation of caspase 3 |
| LOC56899 | | | | -putative 47 kDa protein |
| **LRMP | 12p12.1 | 5′/3′ | 249 | -protein is expressed in a developmentally regulated manner in lymphoid cell lines and tissues |
| PRO1438 | | | | -function unknown |
| KIAA0670 | 14q11.2 | 5′ | 66 | -function unknown, putative DNA binding motif |
| FLJ20671 | | | | -function unknown |
| *CIDEB | 14q11.2 | 5′/cds | 743 | -contains the regulatory CIDE-N domain also in apoptotic pathway proteins CAD and ICAD |
| *BLTR2 | | | | -G protein-coupled receptor, inhibits adenylyl-cyclase, modulates intracellular calcium flux and chemotaxis |
| **ARG2 | 14q24.1 | 3′/cds 3′ | 330 | -catalyzes the hydrolysis of arginine to omithine and urea – located in the mitochondria and expressed in extra-hepatic tissues, especially kidney. |
| *VTI2 | | | | -(v-SNARE); functions in vesicle transport pathways |
| *TK2 | 16q22.1 | 5′+cds/cds | 39 | -generates thymidylate for DNA synthesis |
| FLJ20006 | | | | -function unknown |

Copyright © 2002 John Wiley & Sons, Ltd.

Table 1. Continued

| Name | Map Position | Overlap | Size of Overlap in Nucleotides | Comment |
|---|---|---|---|---|
| *MDDX28* | 16q22.1 | 5′ | 56 | -mitochondrial DEAD-box polypeptide 28 |
| *FLJ20399* | | | | -contains a double-stranded RNA binding domain |
| *LOC51031* | 17p13.3 | 5′+cds | 587 | -function unknown |
| *FLJ10581* | | | | -member of the RNA methyltransferase family |
| *FLJ10534* | 17p13.3 | cds+3′/3′ | 379/397142/142 | -function unknown. |
| **SRR* | | | 80/80 | -catalyses the synthesis of D-serine from L-serine |
| **HUMGT198A* | 17q21.2 | 3′ | 319 | -TBP-1 interacting protein |
| **MLX* | | | | -Interacts with Mad and represses transcription |
| *FLJ10055* | 17q24.2 | 3′ | 539 | -function unknown, contains 2 WD domains |
| *KIAA1001* | | | | -function unknown |
| **MIC1* | 18q11.2 | 3′ | 151 | -colon cancer associated protein |
| ***NPC1* | | | | -integral membrane protein, possible role in cholesterol transport |
| **ERCC-1* | 19q13.3 | 3′ | 112 | -endonuclease |
| **ASE-1* | | | | -may function in rDNA transcription |
| **COL9A3* | 20q13.33 | 3′ | 42 | -alpha 3 subunit of type IX collagen; may connect fibrils to other matrix elements |
| **TCFL5* | | | | -may regulate transcription associated with growth and differentiation |
| ***PPGB* | 20q13.11 | 3′ | 58 | -encodes a glycoprotein – forms a protective complex with beta-galactosidase and neuraminidase for stability and activity. |
| **PLTP* | | | | -phospholipid transfer protein; has roles in phospholipid transport |
| *FLJ10508* | 21q22.3 | 3′+cds/cds | 194/194 | -function unknown |
| **MCM3AP* | | | 159/159 | -protein binds to the replication protein MCM3 |
| **BK126B4.1* | 22q13.2 | cds+3′/3′ | 435/445 | -kraken-like, alpha/beta hydrolase fold |
| *CGI-96* | | | 172/178 | -function unknown,RNA recognition motif |
| **P2RXL1* | 22q11.21 | 3′ | 112 | -member of P2X family of ion channels |
| *SLC7A4* | | | | -NCBI annotation, Cationic amino acid transporter |
| **TR* | | | | -mitochondrial thioredoxin reductase |
| ***COMT* | 22q11.21 | 5′ | 112 | -involved in the degradation of catecholamine neurotransmitters and catechol drugs B membrane bound form. |

[1]5′ UTR of NME7 overlaps with 5′ UTR of BLZF1according to April freeze of the human draft sequence.
[2]3′ of KIAA0764 overlaps with a putative ATP binding protein (NTPBP).
[3]According to April freeze of the human draft sequence, a full overlap exists with matching exons.
[4]SSB originates and terminates within intron of sarcosin, a provisional sequence.
[5]According to the April freeze of the human draft sequence, HSPC070 maps within an intron of RAF-1 along with two other genes B PPARG, which regulates adipocyte and macrophage gene expression and differentiation, and MGC2776, which encodes a hypothetical protein.
[6]Eight alternative transcripts of OGG1, all of which overlap CAMK1. Three of the transcripts overlap the 3′ UTR. The coding region of the other 5 transcripts overlap with CAMK1 coding sequence.

Table 2. Shows the number of pairs of genes that overlapped between the 3′ ends of genes (3′/3′) which are the most numerous, the 5′ ends (5′/5′) or between the 3′end of one with the 5′ end of another gene (3′/5′)

| Type of overlap | Number of overlaps |
|---|---|
| 3′/3′ | 37 |
| 5′/5′ | 11 |
| 3′/5′ | 5 |

nucleotides, in some cases involving the coding region of one transcript (Table 1 and Figure 1).

The transcripts identified in our search encode proteins with heterogeneous functions in DNA synthesis, cell cycle control and developmental regulation. This diversity might suggest that the occurrence of a DNA sequence overlap between pairs of protein-coding genes is incidental to their genomic location and structure, and of no mechanistic significance. However, some of the overlaps detected by our search have previously been reported in the literature, in either human or other species, and include functional studies that support their

Copyright © 2002 John Wiley & Sons, Ltd. 

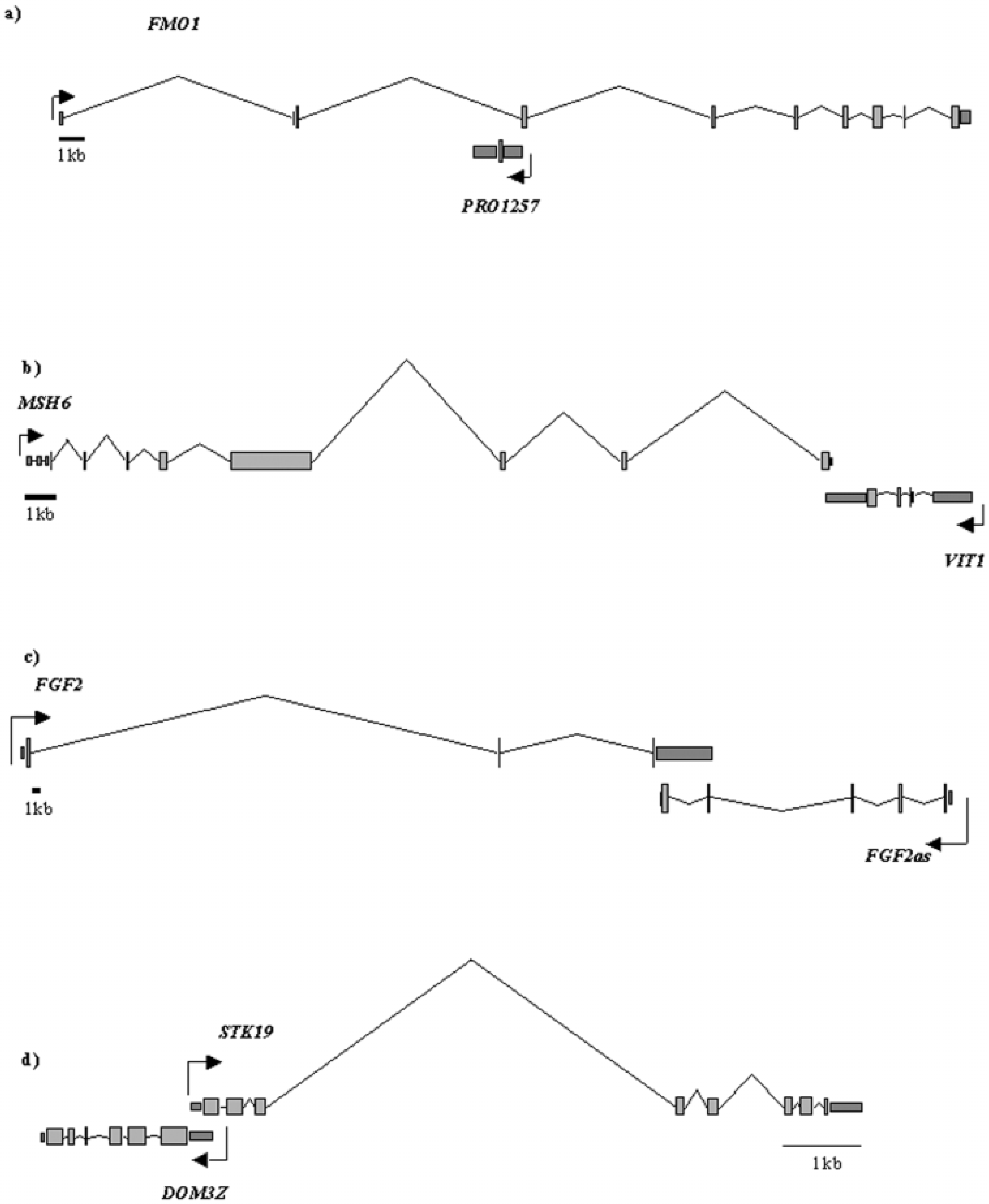

**Figure 1.** Schematic diagrams of some overlapping transcripts found in our search. Lines indicate introns, and boxes exons. a) Overlap between FMO1 and a predicted RefSeq entry on chromosome 1. b) Overlap between the last exon and 3′ UTR of MSH6 and the 3′ UTR of VIT1 on the short arm of chromosome 2. c) Overlap between the 3′ UTR of FGF2 and the coding region and 3′ UTR of its previously reported antisense on the long arm of chromosome 4. d) Overlap between two reviewed RefSeq sequence entries, DOM3Z and STK19, which map to 6p21

Copyright © 2002 John Wiley & Sons, Ltd. 

mechanistic significance. For example, a 1.5 kb OAT to basic fibroblast growth factor (*bFGF*, *FGF2*) has been reported in the oocytes of *Xenopus laevis,* and the human homologue has been cloned and mapped to the long arm of chromosome 4. In *X. laevis*, the region of complementarity extends through both the coding region and the 3′ UTR of *FGF2*, whereas, in the human and rat homologues, complementarity extends only to the 3′ UTR (Figure 1). Expression levels of both the sense and antisense transcripts have been studied to investigate the possibility of antisense regulation of the sense transcript (Li *et al*., 1996). The developmental pattern of expression of the OAT was found to be inversely correlated to the sense transcript in developing rat brain. Expression was also found to be age-dependent with sense expression increasing postnatally and antisense expression decreasing (Li *et al*., 1996). Subsequently, it was shown that FGF2 protein levels are directly influenced by the level of the OAT in mammalian cells (Li *et al*., 2000), suggesting post-transcriptional regulation of FGF2 by the OAT. It has also been shown that this OAT encodes a functional protein with MutT-related enzymatic activity in the rat, and it was noted that the human homologue also contains an open reading frame (Li *et al* 1997).

Intron 3 of the mouse thymidine kinase (*tk*) gene has been reported to contain an antisense promoter and the associated OAT is thought to regulate expression of the TK protein-encoding sense transcript in mouse fibroblasts (Sutterluety *et al*., 1998). This salvage pathway enzyme is expressed at low levels in resting mammalian cells but levels increase dramatically when cells enter S phase. A well-characterized transcriptional regulation is involved, and a post-transcriptional mechanism is also suspected. The correlation of TK protein repression with OAT expression supports a role for the OAT in regulating TK expression. The 5′ UTR and part of the coding sequence of the human *TK* homologue found in RefSeq are complementary to a predicted gene of unknown function indicating the existence of a human homologue of the mouse OAT.

We also found an overlap of 177 nucleotides between the 3′ UTRs of *MSH6* and *VIT1* at 2p16 (Figure 1), as previously reported. It was suggested that the overlap allows regulation of *MSH6* by *VIT1* (Le Poole *et al*., 2000).

Imprinted gene transcripts coding for functional proteins are found in the RefSeq database, but non-coding OATs associated with them are not. For example, the human *COPG2* gene has a non-coding OAT at the 3′ end, which was not found in our search. However, *COPG2* also overlaps with the imprinted, protein-coding *MEST* gene over 52 nucleotides at their 3′ ends, as previously reported (Blagitko *et al*., 1999), and as successfully identified by our search.

In an attempt to identify novel non-coding OATs at imprinted gene loci, a second set of experiments involving a BLASTN search of all known mouse imprinted genes against mouse EST databases found that the majority of imprinted genes had ESTs aligned to both strands. The reverse complement of all gene transcripts in the mouse RefSeq database was also used in a BLASTN search against the same database of mouse ESTs to determine whether the high number of ‘hits’ at imprinted loci occurred as an artefact of the EST database, due to submission of DNA sequence from both strands of cloned, double-stranded cDNA. Out of 7340 entries in mouse RefSeq, 6489 transcripts received hits to the complementary strand. The correct transcriptional orientation of the ESTs aligned to their respective genomic regions could not be assigned unambiguously and therefore the use of EST databases to search for non-coding OATs is unreliable. However, it may be possible in the future to use EST databases consisting exclusively of directionally cloned and sequenced cDNAs to produce an accurate estimate of the frequency of non-coding OATs.

In a further experiment, we assessed the representation of previously confirmed OATs at imprinted mouse and human gene loci in the public EST databases. We found, unsurprisingly, that the databases are biased towards highly expressed transcripts, which is problematic because some OATs are expressed at low levels and may be tissue-specific (Moore *et al*., 1997). The mouse insulin-like growth factor 2 (*Igf2*) gene is an extensively studied imprinted gene with an OAT (*Igf2as*) at the 5′ end (Moore *et al*., 1997; Okutsu *et al*., 2000). This OAT was not detected in our searches, probably due to its low expression level (Figure 2). Moreover, the 5′ ends of genes are underrepresented in EST databases because reverse transcription of mRNA is frequently initiated from the 3′ end using a poly(T) primer. Therefore, in BLASTN searches against ESTs, more ‘hits’ are expected at the 3′ end of the gene (Figure 2). It is also evident that there are many ‘hits’ on the opposite DNA strand to that

Copyright © 2002 John Wiley & Sons, Ltd. 

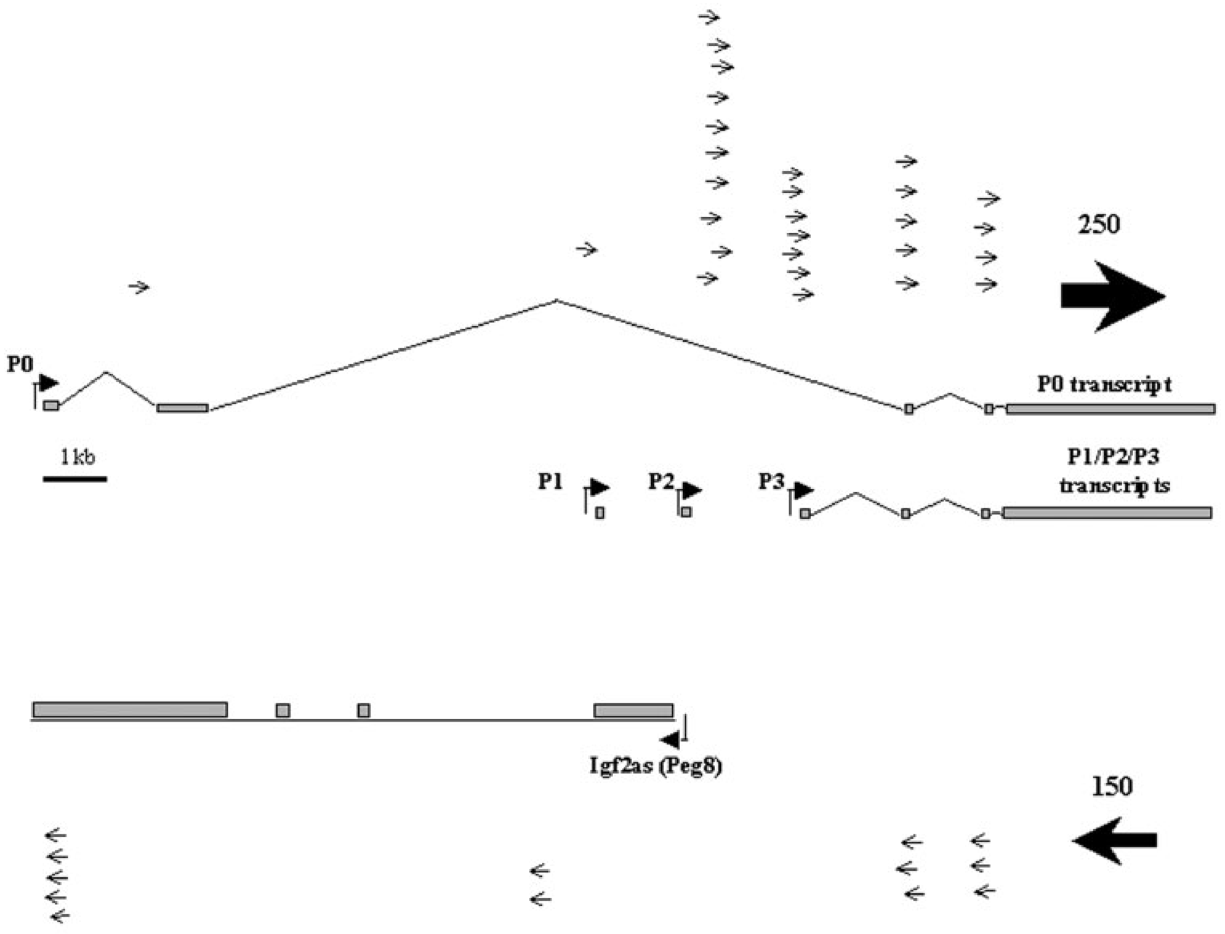

**Figure 2.** Schematic of mouse Igf2 transcripts. Exons are shown as boxes and ESTs aligning to both strands are marked by arrows. EST datasets are biased towards 3′ ends of genes and their orientation with respect to the genomic locus is uncertain. Such bias inhibited the unambiguous validation of non-coding antisense transcripts. Igf2 is one of the most extensively studied imprinted genes with a well-characterised antisense transcript at the 5′ end which was not detected by a search of EST databases. More than 300 ESTs matched both strands at the 3′ end of the gene, as indicated by the thickness of the arrows, with slightly more aligning to the top strand (coding for Igf2) than the lower strand. Relatively few aligned to the 5′ end of the gene predicted from the structure of the *Igf2as* gene, further undermining the reliability of such EST-based searches.

### Discussion

We found 56 pairs of overlapping transcripts among the 11 015 protein coding transcripts in RefSeq. On the conservative assumption that RefSeq contains one quarter of all protein coding transcripts, we can estimate that there are $4 \times 4 \times 56 = 896$ OAT pairs in the human genome. However, this is likely to be an underestimate because RefSeq does not contain non-coding transcripts, which occur frequently at imprinted loci, and also at non-imprinted loci, but at an unknown frequency. In this study, we show that EST data are unsuitable for investigating non-coding OATs due to the biased nature of the current databases. An accurate estimation of OAT pairs consisting of one or two non-coding transcripts will require either laboratory-based approaches or customized gene expression databases that circumvent the problems associated with the current EST databases.

During the preparation of this manuscript a list

Copyright © 2002 John Wiley & Sons, Ltd. 

of potential antisense transcripts in the human genome was reported by Lehner *et al.* (2002). They used RefSeq, as we did, but also used a compilation of vertebrate mRNAs extracted from the EMBL nucleotide sequence database. They reported a total of 87 pairs of genes, 45 of which are in common with our list. Of the 42 gene pairs, reported by Lehner *et al.* (2002) that we did not find, 18 include a sequence from the EMBL compilation that is not represented in RefSeq, and which we did not include in our analysis. These overlapping pairs are of variable and unknown validity but do include some biologically interesting genes. The remaining gene pairs were from a more recent version of RefSeq than that used in our analysis. We report the following 11 unpublished OATs, excluded by Lehner *et al.* (2002) due to the presence of repeat sequences: *TPR/PRG4*, *LOC51611/FLJ20139*, *KIAA0764/FLJ10624*, *CRIPT/LOC51088*, *VRK2/FLJ10335*, *HT009/IDI1*, *APAF1/LOC56899*, *MDDX28/FLJ20399*, *LOC51031/FLJ10581*, *COL9A3/TCFL5*, *FLJ10508/MCM3AP*. However, in all of these pairs, the repeats are not the basis of the complementary pairing between the transcripts. Therefore, as the pairs are transcribed from the same locus their inclusion is valid.

The functional significance of the OATs described herein is largely unknown. However, some of the pairs that we found have been described previously and have been studied functionally (Le Poole *et al.*, 2000; Li *et al.*, 1996, 1997; Sutterluety *et al.*, 1998). Twenty three of the 56 OAT pairs that we describe involve transcripts containing an open reading frame encoding a protein of unknown function. Further characterization of the transcriptome and proteome is required to test the functionality of such pairs. Expression levels of OATs might be expected to be inversely proportional to one another, as is the case for the *FGF2* locus. Such further studies may clarify the involvement of such overlapping transcripts in gene regulation. Although we cannot exclude the possibility that some of the overlaps may be incidental and of no functional significance, the existence of double-stranded RNA specific proteins supports the possibility that OATs constitute part of a significant gene regulatory mechanism. For example, DRADA, a member of the dsRNA-specific adenosine deaminase family of modifying enzymes, is a ubiquitously expressed nuclear enzyme capable of converting adenosine residues in dsRNA molecules to inosines, thereby destabilising the molecule (Kim and Nishikura, 1993). OATs forming dsRNA molecules could also be targets for dsRNA-specific RNases leading to mRNA degradation.

Functionally significant overlapping antisense transcripts have been reported in prokaryotic cells and are implicated in post-transcriptional regulatory mechanisms (Wagner *et al.*, 2002). Regulatory OATs are also present in eukaryotes, indicating a widespread role for antisense mediated gene regulation (Vanhee-Brossollet and Vaquero, 1998). With the emergence of complete genome sequence databases, a comparative analysis to test for interspecies conservation of OAT pairs could offer further insights into the prevalence and functional significance of antisense transcription. For example, the structure of the *FGF2* gene coding transcript and its corresponding OAT are conserved between human, rat, chicken and frog (Knee and Murphy, 1997). This example provides a starting point upon which to build a comprehensive database of OATs. Moreover, as the annotation of genomes becomes more complete, and methods to detect and characterize non-coding transcripts improve, a more complete database of OATs comprising both coding and validated non-coding OATs may be compiled.

### Acknowledgement

This work was funded as part of the Biopharmaceutical Sciences Network funded by the Irish Higher Education Authority. The authors wish to thank Ewan Birney and Toby Gibson for advice at an early stage of this project.

### References

Altschul S, Gish W, Miller W, Myers E, Lipman D. 1990. Basic local alignment search tool. *J Mol Biol* **215**: 403–410.

Blagitko N, Schulz U, Schinzel AA, Ropers H, Kalscheuer VM. 1999. [gamma]2-cop, a novel imprinted gene on chromosome 7q32, defines a new imprinting cluster in the human genome. *Hum Mol Genet* **8**: 2387–2396.

Eddy S. 2001. Non-coding RNA genes and the modern RNA world. *Nat Rev Genet* **2**: 919–929.

Gemund C, Ramu C, Altenberg-Greulich B, Gibson TJ. 2000. Gene2EST: a BLAST2 server for searching expressed sequence tag (EST) databases with eukaryotic gene-sized queries. *Nucleic Acid Res* **29**: 1272–1277.

Gribnau J, Diderich K, Pruzina S, Calzolari R, Fraser P. 2000. Intergenic transcription and developmental remodeling of chromatin subdomains in the human beta-globin locus. *Mol Cell* **5**: 377–386.

Harborth J, Elbashir SM, Bechert K, Tuschl T, Weber K. 2001. Identification of essential genes in cultured mammalian cells using small interfering RNAs. *J Cell Sci* **114**: 4557–4565.

Kim U, Nishikura K. 1993. Double-stranded RNA adenosine

Copyright © 2002 John Wiley & Sons, Ltd. 

deaminase as a potential mammalian editing factor. *Semin Cell Biol* **4**:285–293.

Kiss T. 2001. Small nucleolar RNA-guided post-transcriptional modification of cellular RNAs. *EMBO J* **20**: 3617–3622.

Knee R, Murphy PR. 1997. Regulation of gene expression by natural antisense RNA transcripts. *Neurochem Int* **31**: 379–392.

Kumar M, Carmichael G. 1998. Antisense RNA: function and fate of duplex RNA in cells of higher Eukaryotes. *Microbiol Mol Biol Rev* **62**: 1415–1434.

Le Poole IC, Stennett LS, Sarangarajan R, *et al*. 2000. VIT1, a gene downregulated in Vitiligo Melanocytes, may regulate expression of a G/T mismatch repair protein. Abstract #215 from the 61st annual meeting for the society for investigative dermatology.

Lee JT, Davidow LS, Warshawsky D. 1999. Tsix, a gene antisense to Xist at the X-inactivation center. *Nat Genet* **21**: 343–344.

Lehner B, Williams G, Campbell D, Sanderson CM. 2002. Antisense transcripts in the human genome. *Trends Genet* **18**: 63–65.

Li A, Seyoum G, Shiu R, Murphy PR. 1996. Expression of the rat BFGF antisense RNA transcript is tissue specific and developmentally regulated. *Mol Cell Endocrinol* **118**:113–123.

Li AW, Catherine ATL, Knee R, Wilkinson M, Murphy PR. 1997. FGF-2 antisense RNA encodes a nuclear protein with MutT-like antimutator activity. *Mol Cell Endocrinol* **133**: 177–182.

Li AW, Murphy PR. 2000. Expression of alternatively spliced FGF-2 antisense RNA transcripts in the central nervous system: regulation of FGF-2 mRNA translation. *Mol Cell Endocrinol* **162**: 69–78.

Lukowiak AA, Narayanan A, Li ZH, Terns RM, Terns MP. 2001. The snoRNA domain of vertebrate telomerase RNA functions to localize the RNA within the nucleus. *RNA* **7**: 1833–1844.

Mattick JS. 2001. Non-coding RNA's: the architects of eukaryotic complexity. *EMBO Rep* **2**: 986–991.

McVean GT, Hurst LD, Moore T. 1996. Genomic evolution in mice and men: imprinted genes have little intronic content. *Bioessays* **18**: 773–775.

Moore T, Constancia M, Zubair M, *et al*. 1997. Multiple imprinted sense and antisense transcripts, differential methylation and tandem repeats in a putative imprinting control region upstream of mouse *Igf2*. *Proc Natl Acad Sci U S A* **94**: 12509–12514.

Moore T. 2001. Genetic conflict, genomic imprinting and establishment of the epigenotype in relation to growth. *Reproduction* **122**: 185–193.

Neumann B, Kubicka P, Barlow DP. 1995. Characteristics of imprinted genes. *Nat Genet* **9**: 12–13.

Oktsu TK, Kuroiwa Y, Kagitani F, *et al*. 2000. Expression and imprinting status of human PEG8/IGF2AS, a paternally expressed antisense transcript from the IGF2 locus, in Wilms' tumors. *J Biochem* **127**: 475–483.

Pasquinelli AE, Reinhart BJ, Slack F, *et al*. 2000. Conservation of the sequence and temporal expression of let-7 heterochronic regulatory RNA. *Nature* **408**: 37–38.

Pruitt K, Maglott D. 2001. RefSeq and LocusLink: NCBI gene-centered resources. *Nucleic Acid Res* **29**: 137–140.

Sleutels F, Barlow DP, Lyle R. 2000. The uniqueness of the imprinting mechanism. *Curr Opin Genet Dev* **10**: 229–233.

Smith AFA, Green P. **http://ftp.genome.washington.edu/RM/RepeatMasker.html**.

Sutterluety H, Bartl S, Doetzlhofer A, *et al*. 1998. Growth-regulated antisense transcription of the mouse thymidine kinase gene. *Nucleic Acid Res* **26**: 4989–4995.

Valadkhan S, Manley JL. 2001. Splicing-related catalysis by protein-free snRNAs. *Nature* **413**: 701–707.

Vanhee-Brossollet C, Vaquero C. 1998. Do natural antisense transcripts make sense in eukaryotes? *Gene* **211**: 1–9.

Wagner EG, Altuvia S, Romby P. 2002. Antisense in bacteria and their genetic elements. *Adv Genet* **46**: 361–398.

Wutz A, Smrzka OW, Schweifer N, *et al*. 1997. Imprinted expression of the Igf2r gene depends on an intronic CpG island. *Nature* **389**: 745–749.

Copyright © 2002 John Wiley & Sons, Ltd. 
